# Crimean‐Congo hemorrhagic fever outbreak in Pakistan, 2022: A warning bell amidst unprecedented floods and COVID 19 pandemic

**DOI:** 10.1002/hsr2.1055

**Published:** 2023-01-12

**Authors:** Shehroze Tabassum, Aroma Naeem, Maleeka Zamurad Khan, Nimra Mumtaz, Saima Gill, Laya Ohadi

**Affiliations:** ^1^ School of Medicine King Edward Medical University Lahore Pakistan; ^2^ School of Medicine Shahid Beheshti University of Medical Sciences Tehran Iran

**Keywords:** COVID‐19, Crimean‐Congo hemorrhagic fever, infectious disease, Pakistan, Public health

## Abstract

Crimean‐Congo hemorrhagic fever (CCHF) is an infection caused by a tick‐borne virus (genus: *Nairovirus*, family: *Bunyaviridae*). The most important vector for CCHF is the ixodid tick. Along with tick bite, direct contact with the virus‐affected animal is responsible for its spread. Pakistan witnessed its first case of CCHF in 1976 and has been a major victim of CCHF for years, but spikes in cases are seen after Eid‐ul‐Adha, an Islamic festival involving the sacrifice of cattle. The disease, in particular, is common among butchers, veterinarians, and livestock workers. From the start of this year till June 22, 2022, a total of four cases have been reported across the country. Pakistan faces major challenges in combating CCHF every year due to its specific geographical position and a majority of the population being involved with animal husbandry. There is no approved vaccine for its prevention. All these factors contribute to the burden on the already weakened healthcare system of Pakistan. Strict actions should be taken to contain the spread of the disease. The need of the hour is to engage the general population, raise awareness, and develop policies to ensure disease surveillance. This should be accompanied by fostering collaboration among animal and human health departments for efficient communication and early intervention. The focus should be on medical research to find an efficacious treatment and prophylaxis for the CCHF virus, which will be the cornerstone of future CCHF prevention and control strategies.

## 1 INTRODUCTION

Crimean‐Congo hemorrhagic fever (CCHF) virus is the leading cause of haemorrhagic fever in the world.^1^ It is a tick‐borne viral disease (genus: *Nairovirus*, family: *Bunyaviridae*). It covers a broad geographical area. The name Crimean hemorrhagic fever was derived from its first‐ever case identified in 1944 in the Crimean Peninsula. An important vector and reservoir for CCHF is the ixodid tick, specifically that belonging to the *Hyalomma* genus, having a global spread across more than 40 countries.^2^ This virus has caused multiple hemorrhagic fever outbreaks The incubation period of this virus is 5 or 6 days on average, extending to 13 days Symptoms when evident are sudden in onset, ranging from fever, dizziness, myalgias, sore eyes, photophobia, and headache to agitation, sleepiness, abdominal pain, hepatomegaly, and severe bleeds.^3^ The average mortality rate of this life‐threatening disease is 50%.^4^ Laboratory diagnosis of CCHF can be made by isolation of the virus, immunofluorescence assay, antigen‐capture enzyme‐linked immunosorbent assay (ELISA), reverse transcriptase polymerase chain reaction, and detection of antibody by ELISA (Immunoglobulin G [IgG] and immunoglobulin M [IgM]).^5^ Management of the CCHF virus is mainly supportive. There are no available treatment guidelines according to the severity. Ribavirin is used during the outbreaks.^6^ Pakistan has been a major victim of this infection for years. Tick bites, as well as direct contact with infected animals instantly after slaughter, have been major concerns about the spread of this infection in Pakistan. While CCHF has become endemic to Pakistan, a significant incline in cases is seen in Pakistan in coherence with the arrival of the Islamic festival, Eid‐Ul‐Adha, which involves a sacrifice of the cattle.^7^ Moreover, CCHF has been widespread among butchers, animal husbandry workers, veterinarians, and livestock workers, putting this population at high threat for this disease in CCHF endemic areas of Pakistan.^8^ Domestication of cattle within private houses and the exhibition of flocks of livestock in the markets for sale at and around the time of Eid‐Ul‐Adha has also contributed to the inclination in cases of CCHF in Pakistan.

### 1.1 Epidemiology of CCHF in Pakistan

CCHFV was first discovered in the Russia in Crimean region in 1944, and later in the Congo basin in 1967, earning the virus its present name. More than 50 countries in Asia, Europe, and Africa have reported finding this virus, where it caused either sporadic cases or outbreaks of the disease.^9^ Since its recognition in 1944, the geographic range of CCHFV has grown from the Crimea in the southern Soviet Union to include a sizable area, extending from western China through southern Asia to the Middle East, Bulgaria, the Balkans, as well as most of Africa.^10^

The first ever case of CCHF in Pakistan was reported from General Hospital Rawalpindi in 1976 when a CCHF patient had a laparotomy in the said hospital. It resulted in 11 secondary cases in the hospital and eventually led to the death of 3 people.^11^ This incident resulted in a first‐time report of a high risk of nosocomial transmission of this virus in Pakistan. Later, it was followed by the emergence of multiple cases of CCHF in various provinces of Pakistan. From January 2014 to May 2020, the number of cases of CCHF verified nationwide by the National Institute of Health, Islamabad, Pakistan, is 356, with about a 25% fatality rate. Thirty‐eight percent of these patients were reported from Balochistan, 23% from Punjab, 19% from Khyber Pakhtunkhwa, 14% from Sindh, and 6% from Islamabad.^12^, ^13^, ^14^ According to Zohaib et al. in Pakistan, there is a 2.7% seroprevalence of CCHF, with rural inhabitants having a higher prevalence—probably because they are exposed to more animals field.^15^

**Figure 1:**
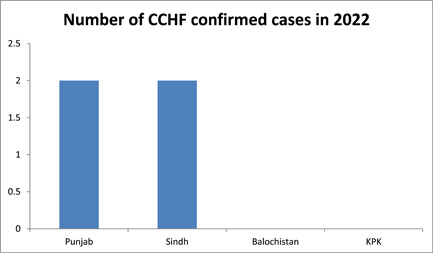
showing the number of CCHF cases confirmed in different provinces of Pakistan in 2022. CCHF, Crimean‐Congo hemorrhagic fever.

The first ever case of CCHF in Pakistan was reported from General Hospital Rawalpindi in 1976 and later was followed by the emergence of multiple cases in various provinces of Pakistan. The disease onset in Pakistan is reported to be sudden, with symptoms beginning on the fourth day of illness and lasting for approximately 2 weeks.^16^ From the start of this year till Jun 22, 2022, a total of four confirmed cases have been reported in Pakistan. Two cases were from the province of Punjab, and two were from the province of Sindh^16^ as shown in Figure 1. Climatic variability in Pakistan can be a causal factor, as reported cases have been more common in March and October.^17^ In recent years, the cases show a decreasing trend. This can be in part due to isolation and preventive measures taken during the COVID‐19 pandemic. Improvement in the infrastructure of health‐system can also be a factor.

### 1.2 CCHF in recent years in Pakistan

Unfortunately, there has been no database for affirmation of the exact number of deaths due to CCHF in Pakistan. In 2016, 22 cases out of 84 suspected patients were confirmed with this disease in Quetta, and 10 out of these 22 cases were reported dead.^18^ In 2018, after the massive outbreak in Karachi, the death toll was reported to be nine.^19^ Karachi reported 20 deaths in 2019.^20^ In 2021, Dr. Sadiq Baloch from Fatima Jinnah Chest and General Hospital, Quetta, confirmed two cases of CCHF, admitted to the hospital.^21^ As of June 22, 2022, four cases have been confirmed in Pakistan, and this has created an alarming situation in Pakistan.^16^

## 2 HURDLES AND CHALLENGES

Since the emergence of the first case of CCHF in Pakistan in 1976, this dangerous infection with a high case mortality rate has become endemic to Pakistan. The increased incidence of CCHF over the years can be attributed to several factors.

### 2.1 Geographical and temporal distribution

CCHF virus is transmitted via tick bite, so the most crucial factor in the equation is the cultivation of tick vectors in an optimal environment. Pakistan provides favorable environmental conditions, particularly a moderate climate, for ticks to grow and multiply.^22^ There is a biannual surge of CCHF infections from March to May as well as from July to September, owing to changing temperatures during these months.^23^ Although infections have been reported from all over the country, Balochistan and Sindh contribute to the most significant number of cases. Balochistan is bordered by Afghanistan and Iran, where CCHF is also endemic. The cross‐border movement of animals and people contributes to the circulation of the CCHF virus in the adjacent regions.^24^ Additionally, the rise in livestock trading, especially during the period of Eid‐ul‐Azha, a Muslim festival where sacrificial animals are slaughtered, leads to the propagation of the virus to far and vast areas of the country. Subsequently, a more significant number of cases are reported during this window.^25^

### 2.2 Animal husbandry

Pakistan is an agricultural country, and most of the rural community engages in rearing livestock. Due to poverty and lack of education, most of the time, livestock do not undergo regular health examinations by veterinary doctors or receive animal vaccines, letting the infection remain unchecked.^26^ Moreover, during Eid‐ul‐Azha, the increased demand for animals further leads to infection outbreaks due to the mishandling of animals and their remains.^25^ Nomadic tribes, moving from place to place with their cattle, are also a source of spreading the infection to a broader population leading to sporadic cases reported outside the endemic regions of the country.^27^

### 2.3 Healthcare system of Pakistan

The healthcare system of Pakistan is not currently equipped to deal with the wide outbreaks of CCHF in the country. There is a lack of proper surveillance systems for early case detection and a scarcity of diagnostic kits for CCHF.^28^ The situation is further complicated by a low index of suspicion among healthcare professionals. According to a study, more than 40% of healthcare professionals have insufficient knowledge about CCHF.^29^ This translates to missed diagnoses and nosocomial spread of CCHF, giving the virus access to a broader population. Even if the diagnosis is made accurately, the provision of quarantine services in hospitals is usually below standard. Lastly, there is a dearth of efficient contact tracing systems in case of lab‐proven infections, leading to rampant virus circulation.^24^

### 2.4 Challenges in management

To date, there has been no approved vaccination for human use to prevent or treat infections from Crimean‐Congo hemorrhagic virus (CCHV).^17^ Once diagnosed, patients are managed with supportive treatment mainly. There is no specific antiviral agent currently developed against CCHV. Ribavirin has shown some benefits, but better pharmacological therapies are still needed to target the CCHF. Several meta‐analyses have ranked the ribavirin trials as low quality as they have been nonplacebo studies. Randomized control trials to establish the efficacy of ribavirin are still missing as it leads to ethical issues. Other experimental drugs like favipiravir, chloroquine, chlorpromazine, and interferon type‐1 have proven efficacy in vitro, but no evidence in humans is available.^1^ A Bulgarian vaccine has been in use since 1974 that has shown some benefits. It is an inactivated vaccine administered subcutaneously prepared from the brain tissue of infected newborn mice. However, international vaccine approval is highly unlikely owing to safety concerns and a lack of efficacy trials. Different approaches have been explored for CCHF vaccine development, including protein antigen targets, but none have been successful yet.^17^

### 2.5 Challenges in diagnosis

Due to similarities in signs and symptoms with other viral diseases, the diagnosis of CCHV might be delayed. Table 1 presents some of the hemorrhagic fevers that resemble CCHV and are strong differentials of CCHF.^30^

**Table 1:** showing differentials of CCHF

Virus Family	Viral Hemorrhagic fevers
Bunyaviridae	Rift valley fever
	CCHV
	HFRS (Hemorrhagic fever with renal syndrome) caused by hanta virus
Flaviviridae	Yellow fever
	Dengue hemorrhagic fever
Arenaviridae	Lassa fever
Filoviridae	Ebola virus disease

Moreover, due to the recent pandemic, the first thing that comes to a physician's mind is COVID‐19. Table 2 provides a comprehensive comparison of different features of CCHV and COVID‐19.^31^, ^32^

**Table 2:** comparing different features of CCHV and COVID‐19

Features	Congo hemorrhagic fever	Covid‐19
Causative agent	Crimean‐Congo hemorrhagic fever virus (CCHFV)	SARS CoV 2
Transmission	It can occur in following ways: By ixoded tick Contact with tissues and fluid of infected animals Nosocomial transmission Sexual transmission Vertical transmission	Through respiratory droplets
Incubation period	5−13 Days	Average 10 (4−14) Days
Clinical Course	Severe	Mild to Severe
Signs and symptoms	Fever Dizziness Myalgias Sore eyes Photophobia Headache Agitation Sleepiness Abdominal pain Hepatomegaly Severe bleeds	Fever and chills Shortness of breath Body aches Headache Malaise Loss of taste and smell Cough Runny nose Diarrhea Nausea and vomiting
Complications	DIC Shock Hemorrhage Multiorgan dysfunction	Shock Respiratory failure Multiorgan dysfunction

## 3 CONGO FEVER IN THE CONTEXT OF RECENT FLOODS IN PAKISTAN

The floods in Pakistan, caused by monsoon rainfall, have impacted more than 33 million people and 75% of the country's districts. Before the floods, Pakistan's public health was already hailed as being inadequate and millions of people now lack access to medical care and treatment. Disease outbreaks like acute watery diarrhea, dengue fever, malaria, polio, and COVID‐19 have been made worse by the crisis, especially in camps and areas where water and sanitation infrastructure has been compromised.^33^ Specifically, in endemic locales, mosquito‐borne diseases like dengue fever may become more common following floods. Standing water could act as a mosquito breeding ground. Therefore, vector‐borne diseases have long‐term effects on overall public health. Rodent‐borne diseases also seem to be more common during periods of heavy rain and flooding.^34^ Climate changes lead to changes in the geographical distribution of vector‐borne diseases and affect the transmission of diseases. One such mechanism is by altering the efficacy of existing interventions.^35^ Similarly, ticks may be drowned by flooding, but the accompanying moist and warm weather can boost tick populations and the danger of tick fever. Therefore, In the aftermath of floods, livestock should be looked out for harboring tick pathogens.^36^ Although there are no definite reports, but the outbreak of Congo fever in the flood‐affected areas with livestock displacement can occur, subsequently leading to a worsening situation for flood‐affected people and overburdening the already fragile healthcare system field.^37^ There is also a high risk of the emergence of a Congo fever outbreak yet again in Pakistan in the flood‐affected areas due to contact with the blood and body fluids of affected livestock which will make the situation worse for flood‐affected people and overburden the healthcare system.^37^ Since the outbreak of cholera^38^ and *N. fowleri* ^39^ has already been recorded in Pakistan in this year, authorities are worried about another Congo fever outbreak amidst these floods.

## 4 RECOMMENDATIONS

### 4.1 Community engagement

Considering that the majority of the cases are seen in veterinarians, farmers, and people working with livestock, community awareness becomes an integral effort to prevent and control the spread of CCHF. This includes appropriate and comprehensible awareness regarding transmission and symptoms of CCHF, safe practices of animal transport, including limiting the contact between livestock and humans, using tick repellent, regular skin and clothing examination for ticks, and limiting the consumption of unpasteurized milk and uncooked meat.^40^ In areas where subsistence farming is practiced, people dealing with cattle usually lack resource mobilization and awareness, so unique methods should be employed, including airing on commonly used platforms, radio, and television in their native language. Near Eid‐ul‐Adha, mass awareness about safe waste disposal and slaughter practices should be promoted. Further, people should be encouraged to contact the health care setup in case of symptoms immediately.

### 4.2 Improvement of healthcare infrastructure and disease surveillance

In Pakistan, nosocomial spread, in addition to sporadic community‐contracted disease, has been reported. This points towards the need for the mobilization of resources towards improved isolation facilities, early diagnosis, efficient contact‐tracing system, and training of healthcare professionals regarding early diagnosis and strict adherence to standard infection control measures in a hospital setting. The provision of better diagnostic capabilities and contact tracing will be of paramount importance in preventing an epidemic. Ironically, laboratory facilities are minimal in remote rural areas where the cases originated. So, attention must be given to increasing the laboratory and diagnostic capacity in endemic, high‐risk areas. It must be ensured that disease surveillance mechanisms are being employed and working efficiently nationwide. The only imprudent approach in a developing country like Pakistan is relying upon analysis of individual cases in relation to their locality, prevalence, and fatality, as there is no appropriate pattern of mortality, disease surveillance, and data pool of confirmed cases in Pakistan. Baluchistan, an underdeveloped province of Pakistan, lacks a surveillance system for early disease detection; however, a new initiative has been introduced in Khyber Pakhtunkhwa Province, named Integrated Disease Surveillance and Response System (IDSRS), which aims to detect disease earlier for prompt treatment.

### 4.3 Animal surveillance

Routine animal surveillance for tick infestation is recommended, and strategies for managing tick infestation, including identifying host animals, pesticide use, and controlling tick breeds, should be initiated. This is particularly important near Eid‐ul‐Adha, where assessment in animal markets can prevent spill‐over of CCHF in humans.

### 4.4 Intersectoral collaboration

A collaboration among animal and human health sectors will prevent tick‐human, animal‐human, and human‐to‐human transmission. This can prevent potential havoc on the already burdened healthcare system of Pakistan.

### 4.5 Cross‐border collaborations and policies

Strengthening surveillance of animals and people traveling from disease‐endemic countries should be ensured, which would play a vital role in the control of the outbreaks due to transmission from neighboring endemic countries like Iran.

### 4.6 Promoting medical research

CCHF is listed by World Health Organization (WHO) as one of the top priority diseases for research and development in the public health emergency context.^41^ This area needs extensive research in the fields of diagnosis, vaccination, and treatment. The time between the onset of nonspecific symptoms and confirmed CCFH is a time for potential nosocomial and close contact spread. The utilization of faster diagnostic tests for CCHF in primary care settings, standardization of case definitions, and development of algorithms for early triage will be keystones in preventing disease in its early stages. There is no approved vaccine for CCHF. Future CCHF control strategies must work on this front.

Furthermore, collaboration among researchers worldwide must be ensured for targeted, demographically specific vaccine provision. Regarding treatment, randomized control trials to confirm the efficacy of ribavirin should be conducted. Antivirals with proven efficacy for CCHFV should be developed.

### 4.7 Counteractive measures during recent floods in Pakistan

To properly tackle the situation and prevent its spread to other parts of the country, certain measures must be taken including making operational case definitions for suspected, probable, and confirmed cases to assist decision‐making on the ground, guidelines for the outbreak management team to detect, identify, verify and report the cases in time, strategies for health service providers to manage, prevent and raise awareness in the community about CCHF disease and establishment of an efficient reporting and surveillance system. To make the efforts fruitful, every individual needs to identify and fulfill their responsibility and cooperate with the National public health teams to control and prevent CCHF in Pakistan.

## AUTHOR CONTRIBUTIONS

**Shehroze Tabassum**: Conceptualization; writing – original draft; writing – review and editing. **Aroma Naeem**: Writing – original draft; writing – review and editing. **Maleeka Zamurad Khan**: Writing – original draft. **Nimra Mumtaz**: Writing – original draft. **Saima Gill**: Writing – original draft. **Laya Ohadi**: Writing – original draft.

## TRANSPARENCY STATEMENT

The lead author Laya Ohadi affirms that this manuscript is an honest, accurate, and transparent account of the study being reported; that no important aspects of the study have been omitted; and that any discrepancies from the study as planned (and, if relevant, registered) have been explained.

## Data Availability

Data sharing not applicable–no new data generated.
